# Evaluation of a commercial multispecies rapid test for anti-*Leishmania infantum* antibodies in unconventional animal species

**DOI:** 10.1007/s11259-026-11117-3

**Published:** 2026-02-28

**Authors:** Sergio Villanueva-Saz, Ana González, María D. Pérez, Delia Lacasta, Antonio Fernández, Pablo Quilez, Aurora Ortín, David Guallar, Álex Gómez, Diana Marteles-Aragüés

**Affiliations:** 1 Department of Animal Pathology, Veterinary Faculty, University of Zaragoza, 177 Miguel Servet Street, 50013 Zaragoza, Spain; 2 Clinical Immunology Laboratory, Veterinary Faculty, University of Zaragoza, Zaragoza, 50013 Spain; 3 Instituto Agroalimentario de Aragón-IA2 (Universidad de Zaragoza-CITA), Zaragoza, 50013 Spain; 4 Department of Animal Production and Food Science, Veterinary Faculty, University of Zaragoza, Zaragoza, 50013 Spain

**Keywords:** Animals, ELISA, Leishmania infantum, Immunochromatographic test, Point-of-care test, Serology

## Abstract

*Leishmania infantum* is a vector-borne protozoan causing zoonotic visceral leishmaniasis in the Mediterranean basin, where dogs are the primary reservoir. However, infection in various domestic and wild mammals raises questions about their epidemiological roles. Reliable diagnosis in non-canine species remains challenging, as most serological assays are developed for dogs and cats. This study evaluated a commercially available multispecies rapid immunochromatographic test (Uranotest^®^ *Leishmania* feline) for detecting anti-*Leishmania* antibodies in a diverse panel of domestic and non-domestic animals. A total of 186 serum samples from different species were analyzed, previously classified as seropositive (*n* = 65) or seronegative (*n* = 121) using an in-house ELISA as reference. Immunochromatographic rapid test performance was compared to ELISA results using Cohen’s kappa to assess agreement. Overall agreement reached 82.3% (κ = 0.56), indicating moderate concordance. This immunochromatographic test correctly identified all positive and negative canine and feline samples and showed perfect agreement in other carnivores such as mink and wolves. In contrast, all herbivorous species, including alpaca, horses, goats, llama, sheep, bison, and dromedaries, produced negative results by the rapid test despite ELISA seropositivity, suggesting limited affinity of the conjugate for herbivore immunoglobulins. These findings indicate that, while the assayed rapid test is suitable for detecting anti-*Leishmania* antibodies in non-herbivorous species, it is unreliable for herbivores. The study highlights the importance of species-specific validation of serological assays and suggests that rapid tests using protein A conjugates may have restricted cross-species utility, reinforcing the need for adapted diagnostic tools in multispecies and wildlife contexts.

## Introduction

*Leishmania infantum* is a vector-borne protozoan of major veterinary and public health relevance in the Mediterranean basin. Transmitted primarily by phlebotomine sand flies, *L. infantum* causes a spectrum of clinical outcomes ranging from subclinical infection to severe systemic disease in humans. While domestic dogs are widely recognized as the principal reservoir for human zoonotic visceral leishmaniasis (Moreno and Alvar 2002), increasing evidence indicates that infection also occurs in a broad array of non-domestic mammals, including wild canids (Millán et al. 2014), mustelids (Villora et al. 2025), felids (Cavalera et al. 2020), lagomorphs (Fernández-Arévalo et al. 2024), and captive exotic species (Villanueva-Saz et al. 2025; Barbero-Moyano et al. 2026). The epidemiological role of other hosts different from dog and cat is not yet fully elucidated; however, their exposure to vectors and the frequent detection of *L. infantum* DNA (Merino Goyenechea et al. 2023) or specific antibodies (Giner et al. 2022; Cantos-Barreda et al. 2020; Moraleda-Berral et al. 2025) against *L. infantum* suggests that they may contribute to local transmission cycles or serve as sentinels of parasite circulation.

Diagnosis of *L. infantum* infection in animals can be approached through parasitological, molecular, and serological methods (Villanueva-Saz et al. 2021). Direct parasitological techniques such as cytology, histopathology, and parasite culture offer high specificity but are limited by variable sensitivity. Molecular assays, provide sensitive detection of parasite DNA across different tissues and are especially valuable for early infection or in cases with low antibody responses. Nonetheless, molecular methods require laboratory infrastructure, standardized sampling protocols, and careful interpretation, especially in wildlife studies where sample quality and pathogen load may be heterogeneous (Millán et al. 2014).

A critical analytical constraint in multispecies serology is the interaction between assay conjugates and host immunoglobulins. Protein A, protein G, and hybrid reagents (protein A/G) exhibit heterogeneous binding affinities across mammalian taxa, which can translate into species-dependent sensitivity in both ELISA and lateral flow formats. Therefore, conjugate choice is a key determinant of cross-species applicability of rapid immunochromatographic tests.

Serology remains one of the most widely used approaches in epidemiological surveys and clinical practice because it is relatively accessible, can be performed on serum or plasma, and supports large-scale screening (Barbero-Moyano et al. 2025). Several serological techniques have been applied to detect anti-*Leishmania* antibodies in animals. Indirect immunofluorescence antibody test (IFAT) has historically been a reference method in dogs and is frequently adapted for other species (Giner et al. 2020; Barbero-Moyano et al. 2025; Murillo et al. 2026). However, it requires specialized equipment and trained personnel, and species-specific conjugates are often needed for reliable results. Enzyme-linked immunosorbent assays (ELISA), offers high-throughput capacity and can be optimized for sensitivity and specificity. ELISA technique performance may be influenced by cut-off definition, and interspecies differences in immune responses (Barbero-Moyano et al. 2025). Finally, Western Blot (WB) can provide detailed antibody profiling and may help resolve discordant cases (Marteles et al. 2024; Murillo et al. 2026), but it is time-consuming and less practical for routine or large-scale use.

A key challenge in applying serology to non-domestic species is the lack of validated, commercially standardized assays. Many protocols rely on in-house adaptations of canine tests, often with limited sample sizes and without species-specific performance metrics. Importantly, rapid immunochromatographic tests widely used in dogs due to their practicality and speed, are generally designed and validated for canine antibodies, and their diagnostic accuracy may not translate reliably to other species (Villanueva-Saz et al. 2022). This diagnostic gap hinders clinical decision-making in zoological medicine and limits surveillance capabilities in wildlife.

In general, serological techniques must be adapted to the animal species, first, for example, using different conjugate for the serological technique and then validated to evaluate diagnostic measures including sensitivity and specificity (Villanueva-Saz et al. 2021). In this context, the development of a multispecies rapid test capable of detecting anti-*Leishmania* antibodies across a range of other animals represents a significant advance; adaptation to each species constitutes the first step in the development of serological techniques.

Given the scarcity of commercially available rapid tests validated beyond dogs and cats, this study aimed to evaluate a multispecies rapid immunochromatographic test for detecting anti-*Leishmania* antibodies in a range of domestic and non-domestic species.

## Materials and methods

### Serum sample selection

One hundred eighty-six serum samples were selected from the sera collection of Clinical Immunology Laboratory (University of Zaragoza, Spain). Samples were submitted to the laboratory for various diagnostic purposes, including annual screening of clinically healthy animals and investigation of suspected clinical leishmaniosis. These samples were submitted from June 2022 and December 2025. Two aliquots of each sample were prepared and stored at − 20 °C until testing. In this sense, recent evidence indicates that long-term freezing at − 20 °C–− 80 °C preserves anti-*Leishmania* IgG, supporting retrospective serology (Marteles et al. 2025; Olmeda et al. 2025). For this study, samples with different anti-*Leishmania* IgG status were included, comprising a panel of seropositive animals and a panel of seronegative samples based on a multispecies in-house ELISA (Barbero-Moyano et al. 2025).

Seropositive samples were divided into two groups. The first group consisted of samples from clinically ill animals with confirmed *L. infantum* infection and included: Bennett’s wallaby (*n* = 4), european mink (*n* = 7), goat (*n* = 1), horse (*n* = 1), tiger (*n* = 1) and white-naped mangabey (*n* = 1). The second seropositive group comprised samples from seropositive animals of the following species: alpaca (*n* = 1), american mink (*n* = 1), european bison (*n* = 4), dromedary (*n* = 4), horse (*n* = 6), iberian wolf (*n* = 1), llama (*n* = 1), rabbit (*n* = 3), and sheep (*n* = 10). As seropositive control, a panel of sick cats (*n* = 6), sick dogs (*n* = 6) and seropositive cats (*n* = 7) were included in the study.

In addition, a panel of seronegative samples was included. These samples belonged to animals classified as clinically healthy based on veterinary examination and were as follows: alpaca (*n* = 4), american mink (*n* = 10), Bennett’s wallaby (*n* = 10), dromedary (*n* = 10), european bison (*n* = 10), european mink (*n* = 10), goat (*n* = 10), horse (*n* = 10), iberian wolf (*n* = 1), llama (*n* = 4), rabbit (*n* = 10), sheep (*n* = 10), tiger (*n* = 1), and white-naped mangabey (*n* = 1). Finally, as negative controls, a panel of seronegative non infected cats (*n* = 10) and dogs (*n* = 10) were included.

## Detection of leishmania infantum antibodies by immunochromatographic rapid test (ICT)

The rapid test (Uranotest^®^*Leishmania* feline, Uranovet, Spain) was performed following the instructions of the manufacturer. All tests were stored at room temperature and were performed as described in the instructions supplied with the test kit. The diagnostic kit is based on the immunochromatographic technique and is designed for the qualitative determination of antibodies against *L. infantum* in feline whole blood, serum, or plasma. This ICT uses protein A as a capture reagent for the rapid detection of *L. infantum*-specific antibodies and may be applicable to animal species in which protein A exhibits affinity for immunoglobulin G (IgG) (Villanueva-Saz and Marteles 2024). The test consists of a reactive strip containing a rounded well where the sample is added and a results area that contains the T line (test line) and the C line (control line). Once the sample is applied to the rounded well, migration by capillary action begins along the membrane. If the result is negative, a single purple band will appear in the C area. The band in the C area must always appear, as it is a control band indicating that the test has been performed correctly. If the result is positive, in addition to the C band, a purple band will appear in the test area (T line). Briefly, 10 µL of serum was added to the sample window of the test device, followed by 2 drops (60 µL) of the buffer provided in the kits. The test was read 10 min after the addition of the sample. All tests were read by two laboratory technicians after 10 min. If discrepancies arose between results, a third observer participated. The examiners were blinded to the results of the quantitative serological tests. ICT was performed in duplicate in all samples included in the study.

## Detection of Leishmania infantum antibodies by in-house ELISA

The presence of antibodies against *L. infantum* was determined using an in-house ELISA (sensitivity of 99.4% and specificity of 97.5% by latent class analysis), as previously described (Barbero-Moyano et al. 2025). In this ELISA, 100 µL of Protein A/G conjugated to horseradish peroxidase diluted 1:10,000 was added per well. This reagent interacts with immunoglobulin G in different mammal species, allowing the use of positive and negative controls from different species in the absence of controls for the species being serologically tested. The cut-off for carnivores was set to 0.220 optical density value (OD), while the cut-off for herbivores was set to 0.380 OD. By contrast, species-specific cut-off values were set for the american mink, european bison, european mink, goat, horse, sheep, and white-naped mangabey (Table 1). Sera were classified as high positive when they had an OD equal to or higher than 1.5. Medium positive sera were classified as having an OD equal to or higher than 0.7 but less than 1.5. Finally, low positive sera were defined as those with an OD lower than 0.7 but higher than the cut-off.

**Table 1:** Species-specific ELISA and rapid test results in seropositive animals

Animal Group (number of animals)	Animal Species	Number of samples included	ELISA Seropositivity classification (Optical density value, OD)	ICT result	ELISA Cut-off
Sick animal (*n* = 27)	Bennett’s wallaby (*Macropus rufogriseus rufogriseus*)	4	High (2.646)	-	≥ 0.380
			High (3.910)	-	
			High (1.725)	-	
			Medium (0.976)aw	-	
	Cat (*Felis catus*)	6	High (2.246)	+	≥ 0.130
			High (2.227)	+	
			High (2.176)	+	
			Medium (1.316)	+	
			Medium (1.248)	+	
			Medium (1.230)	+	
	Dog (*Canis lupus familiaris*)	6	High (3.375)	+	≥ 0.220
			High (1.789)	+	
			High (1.675)	+	
			Medium (1.436)	+	
			Medium (1.071)	+	
			Medium (0.997)	+	
	European mink (*Mustela lutreola*)	7	High (3.561)	+	≥ 0.200
			High (3.554)	+	
			High (3.562)	+	
			High (3.584)	+	
			High (3.352)	+	
			High (1.883)	+	
			High (1.714)	+	
	Goat (*Capra aegagrus hircus*)	1	Medium (1.324)	-	≥ 0.260
	Horse (*Equus caballus*)	1	Medium (0.560)	-	≥ 0.300
	Tiger (*Panthera tigris*)	1	Medium (1.018)	+	≥ 0.220
	White-naped mangabey (*Cercocebus lunulatus*)	1	Medium (1.211)	+	≥ 0.200
Seropositive animals (*n* = 38)	Alpaca (*Vicugna pacos*)	1	Low (0.569)	+	≥ 0.380
	American mink (*Neogale vison*)	1	High (1.590)	+	≥ 0.200
	Cat (*Felis catus*)	7	High (3.594)	+	≥ 0.130
			High (3.2421)	+	
			High (1.983)	+	
			High (1.796)	+	
			Medium (1.145)	+	
			Medium (1.035)	+	
			Medium (0.959)	+	
	European Bison (*Bison bonasus*)	4	Low (0.657)	-	≥ 0.330
			Low (0.525)	-	
			Low (0.493)	-	
			Low (0.423)	-	
	Dromedary (*Camelus dromedarius*)	4	High (1.936)	-	≥ 0.380
			Medium (0.811)	-	
			Medium (0.742)	-	
			Low (0.637)	-	
	Horse (*Equus caballus*)	6	Medium (1.628)	-	≥ 0.300
			Medium (1.308)	-	
			Medium (0.730)	-	
			Medium (0.703)	-	
			Low (0.632)	-	
			Low (0.606)	-	
	Iberian Wolf (*Canis lupus signatus*)	1	High (1.620)	+	≥ 0.220
	Llama (*Lama glama*)	1	Low (0.429)	+	≥ 0.380
	Rabbit (*Oryctolagus cuniculus*)	3	Medium (1.101)	-	≥ 0.380
			Medium (1.430)	-	
			Medium (1.320)	-	
	Sheep (*Ovis orientalis aries*)	10	High (2.205)	-	≥ 0.380
			Medium (1.553)	-	
			Medium (1.227)	-	
			Medium (1.161)	-	
			Medium (1.120)	-	
			Medium (1.044)	-	
			Medium (0.969)	-	
			Medium (0.956)	-	
			Medium (0.935)	-	
			Medium (0.843)	-	

Abbreviations: + positive, - negative

### Statistical analysis

Data collected were analysed using descriptive statistics (IBM^®^ SPSS^®^ Statistics software version 29, SPSS Inc. Chicago, IL, USA). The agreement between serological diagnostic techniques and each species was evaluated by the use of kappa index. The kappa agreement between serological diagnostic techniques was determined as follows: no agreement (k < 0), slight agreement (0 < k < 0.2), fair agreement (0.2 < k < 0.4), moderate agreement (0.4 < k < 0.6), substantial agreement (0.6 < k < 0.8) and almost perfect agreement (k > 0.8).

## Results

### ICT results

The performance of the ICT was evaluated against a panel of negative and positive sera previously classified by the in-house ELISA as reference technique. A total of 65 animal serum samples were evaluated, divided into sick animal (*n* = 27) and seropositive animals (*n* = 38), with species-specific ELISA cut-offs ranging from ≥ 0.130 to ≥ 0.380. In the sick animal group, high-to-medium ODs were observed particularly in European mink (*n* = 7), and this species also showed uniform ICT positivity for all listed samples. The tiger (*n* = 1) and white-naped mangabey (*n* = 1) were also ICT positive with medium ELISA ODs. In contrast, Bennett’s wallaby (*n* = 4), goat (*n* = 1), and horse (*n* = 1) in this group showed negative ICT results despite medium-to-high ELISA classifications in some cases. In the seropositive animal group, ICT positivity was again seen in select carnivores, including American mink (*n* = 1), cats (*n* = 7), and Iberian wolf (*n* = 1), with ELISA ODs ranging from medium to high. However, several species with low-to-medium ELISA ODs including alpaca (*n* = 1), European bison (*n* = 4), dromedary (*n* = 4), horse (*n* = 6), llama (*n* = 1), rabbit (*n* = 3), and sheep (*n* = 10), were consistently ICT negative (Table 1). Finally, samples included as part of the positive and negative panel controls were correctly classified by the ICT.

Using the binary classification, we assessed agreement between ELISA and the ICT across 186 serum samples. Overall observed agreement was 82.3% (153/186), yielding a Cohen’s kappa of 0.56 (moderate agreement; 95% CI 0.43–0.68). The global 2 × 2 distribution was ELISA⁺/ICT⁺ = 32, ELISA⁺/ICT⁻ = 33, ELISA⁻/ICT⁺ = 0, and ELISA⁻/ICT⁻ = 121, indicating that discordance was driven primarily by ELISA-positive samples that were ICT-negative. Importantly, no ELISA⁻/ICT⁺ results were observed (0/121), supporting the absence of ICT false positives and therefore a high specificity of the rapid test in the evaluated dataset. Species-level kappa values showed two distinct patterns: perfect agreement (k = 1.00) in felids, canids, and minks included in the table, and k = 0.00 in several ungulates and lagomorphs. The latter should not be interpreted as random lack of concordance, but rather as a structural pattern of unidirectional disagreement whereby ELISA-positive animals consistently tested ICT-negative.

## Discussion

This study provides the first evaluation of a commercially available multispecies rapid test for the detection of anti-*Leishmania* antibodies in a panel of unconventional animal species. There is limited information regarding the application of ICT to species other than dogs to detect the presence of anti-*Leishmania* antibodies. In red foxes and cats, an in-house ICT based on Protein A conjugated showed good concordance with established serological reference techniques (Anfossi et al. 2018). More recently, a commercial ICT (SNAP^®^ Leish 4Dx^®^, IDEXX Laboratories Inc.) for use in dogs was able to detect the presence of anti-*Leishmania* antibodies in captive lemurs (*Lemur catta*) (Fagundes-Moreira et al. 2025).

Serological tools remain essential for *L. infantum* diagnosis and surveillance because they are practical, scalable, and less invasive than parasitological methods. However, extending serological assays beyond dogs is challenging due to interspecies differences in immune responses and, importantly, differences in test chemistry compatibility. Whereas the rapid test detected seropositive samples in several non-herbivorous species, all seropositive herbivore samples yielded negative results. This pattern suggests that the test uses a single cut-off value specifically defined for one species (feline, a carnivore), but its potentially multispecies design is likely influenced by the type of conjugate employed in the assay (Thermo Fisher Scientific 2013) and should therefore be interpreted with clear, species-specific caution.

This study provides the first evaluation of the performance and practical impact of a commercially available rapid test for the detection of anti-*Leishmania infantum* antibodies in non-canine species. The inclusion of multiple taxonomic groups and clinically relevant hosts supports the potential applicability of this tool beyond dogs and helps address a clear diagnostic gap in zoological, wildlife, and mixed-species settings. A further strength is the use of a well-characterized panel of seropositive and seronegative samples, allowing an initial assessment of agreement between the rapid test and the reference serological classification.

Interpretation of k in this study requires caution because k value is sensitive to prevalence and to marginal distributions. The predominance of concordant ELISA⁻/ICT⁻ samples increases the observed agreement, while k may underestimate agreement in settings with highly unbalanced margins. In practical terms, the dataset shows a clear asymmetry: discordance was unidirectional (ELISA⁺/ICT⁻) and no ICT false positives were recorded, which supports high specificity of the ICT across the species included. Conversely, k = 0.00 in herbivores does not indicate stochastic disagreement but a systematic failure to detect ELISA-positive sera with the ICT, consistent with limited affinity of the ICT conjugate for herbivore IgG. Therefore, k values should be read alongside the 2 × 2 table and the direction of discordance when comparing performance across taxa.

Several limitations should be considered when interpreting these findings. First, the sample size for some species was small, which limits the precision of species-specific performance estimates and prevents robust calculation of sensitivity and specificity by host category. Second, because this was a retrospective panel-based evaluation, samples may not fully represent the diversity of infection stages, antibody titers, or clinical phenotypes encountered in field conditions. Third, serological classification was used as the reference framework; therefore, the study does not directly address how rapid test results correlate with parasitological or molecular confirmation across all species. Unlike in dogs, where samples covering a wide range of antibody titers are readily available (Villanueva-Saz et al. 2022), access to diverse serological profiles is limited in other species, including cats (Alcover et al. 2021). As a result, this evaluation primarily assessed the test’s ability to detect clearly seropositive samples, rather than its performance across the full spectrum of titers.

Future work should evaluate alternative conjugate strategies to extend performance to herbivores, including protein G (or protein L, depending on isotype binding requirements), optimized protein A/G formulations, or species-specific anti-IgG conjugates when feasible. A practical development pathway could combine targeted conjugate selection informed by known Fc-binding profiles across taxa with species-level validation panels to estimate sensitivity and specificity and to define appropriate cut-offs.

In conclusion, this study addresses a significant diagnostic gap by evaluating a commercial rapid test in non-canine species. The discordance observed in herbivores appears to be primarily driven by low conjugate affinity for herbivore antibodies, underscoring a key technical limitation of the current assay format. These findings reinforce the importance of species-specific validation.

## Data Availability

Not applicable.
